# The effect of biochar prepared at different pyrolysis temperatures on microbially driven conversion and retention of nitrogen during composting

**DOI:** 10.1016/j.heliyon.2023.e13698

**Published:** 2023-02-13

**Authors:** Haihou Wang, Tianyun Shao, Yujie Zhou, Xiaohua Long, Zed Rengel

**Affiliations:** a Suzhou Academy of Agricultural Sciences, Institution of Agricultural Sciences Taihu Lake District, Suzhou, 215155, China; b College of Resources and Environmental Sciences, Nanjing Agricultural University, Nanjing, 210095, China; c Soil Science and Plant Nutrition, UWA School of Agriculture and Environment, The University of Western Australia, 35 Stirling Highway, Perth, WA, 6009, Australia; d Institute for Adriatic Crops and Karst Reclamation, Put Duilova 11, Split, Croatia; e National Soil Quality Observation and Experimental Station in Xiangcheng, Suzhou, 215131, China

**Keywords:** Agricultural residues, Biochar, Nitrogen conversion, Nitrate nitrogen, Nitrogen loss rate

## Abstract

Aerobic composting is one of the most economical ways to produce organic fertilizer from agricultural wastes. In this research, we independently developed a simple composting simulation reactor. The effects of biochar pyrolysised at different pyrolysis temperatures (B1-450 °C; B2-550 °C; and B3-650 °C) on nitrogen conversion (Total nitrogen (TN), ammonium nitrogen (NH_4_^+^-N), nitrate nitrogen (NO_3_^−^-N), cumulative amount of ammonia (CEA) and nitrous oxide (CEN) emission, nitrogen loss rate (NLR), etc.) and functional microbial community (*cbbL*, *cbbM* and *nifH*) structure in the composting system were studied. Results showed that the addition of biochar significantly improved the efficiency of composting, increased the NO_3_^−^-N concentration and reduced the NLR (%) in the composting system (B3 (31.4 ± 2.73)<B2=B1 (41.7 ± 3.29)<B0 (54.5 ± 3.34), *p* ≤ 0.05), while the loss rate of nitrogen positively correlated with compost pH. Denitrifying bacterial genera such as *Pseudomonas, Alcaligenes, Paracoccus, Bacillus, Citrobacter, Mesorhizobium, Thiobacillus* and *Rhodococcus* in this study was an important reason for nitrogen loss during composting, and the abundance of autotrophic microorganisms (such as S*ulfuritalea, Hydrogenophaga, Thiobacillus, Thiomonas* and *Candidatus_Thioglobus*) in treatments with biochar (B1, B2 and B3) were higher than that in B0. Besides, the community structure in the treatments B2 and B3 was similar at the end of composting and clearly distinguished from that in B1. Moreover, the five functions predicted by OTUs in this study with the highest proportions were chemoheterotrophy, nitrate reduction, fermentation, aerobic chemoheterotrophy and nitrogen respiration. The study provided a theoretical basis for the application of biochar to improve the compost-related processes.

## 1 Introduction

The large-scale and intensive development of agriculture, livestock and poultry industry has produced a large amount of agricultural residues and waste, resulting in a number of ecological and environmental problems, including those that threaten human health [[1], [2], [3]]. Aerobic composting is one of the most economical and feasible ways to turn agricultural residues to organic fertilizer [4,5].

Decomposing organic waste into useable organic fertilizer through high-temperature aerobic fermentation can achieve not only the regeneration and recycling of materials in the agricultural ecosystems, but also underpin the use of composted organic products in replacing chemical fertilizers to meet the need for reduced use of chemical fertilizers. Moreover, this process can effectively eliminate harmful microorganisms and weed seeds in dung [6,7].

However, large nitrogen losses are common in composting. The loss of nitrogen is about 19%–77% of the initial total nitrogen [[8], [9], [10]]. This not only reduces the agricultural value of the compost, but also causes environmental problems because of the release of NH_3_, N_2_O and NO, etc. Therefore, reducing nitrogen loss in composting has become a key technical requirement. Strengthening the fixation and retention of nitrogen during composting is the key to promoting a virtuous synergism of agriculture, rural ecology and economy [[11], [12], [13]]. The determination of nitrogen concentration in compost is one of the most important factors when studying its agronomic value.

Generally, biochar (BC) is a highly aromatic porous solid material with strong adsorption ability produced by pyrolysis of biomass waste at relatively low temperatures (<700 °C) [14,15]. In recent years, the application of biochar has become an emerging technology in environmental use. As an auxiliary composting material, biochar has been used as an amendment to improve the compost quality and to shorten the maturity process during the composting process since recently [16,17], and the above-mentioned effects of BC for storing moisture and nutrients also mean that the biomass waste is better degraded microbiologically. Many reports have proved that BC could be used as amendments to the nitrogen assimilation ability of microorganisms and to reduce nitrogen loss in composting [[18], [19], [20], [21]], to enhance microbial activity [14,16,22] and to facilitate the composting process, to decrease the water-soluble carbon content and to reduce the mobility of heavy metals [17,18,23]. Besides, researchers proposed a sustainable biochar concept, through which the emission of greenhouse gases including nitrous oxide (N_2_O) and methane (CH_4_) can be avoided [[24], [25], [26], [27]].

However, the pyrolysis temperature is strongly correlated with changes in the structure and physicochemical properties (e.g. surface area, pH and functional groups) of BC [22,[28], [29], [30]], and it affected on the functions of BC [31,32]. Moreover, the type and concentration of surface functional groups have been reported to play an important role in adsorption capacity and the removal mechanism of adsorbates by BC [33]. Ghani et al. [34] have shown that at lower temperatures (<500 °C), BC becomes more hydrophilic, and BC is thermally stable and becomes more hydrophobic at 600–700 °C [[34], [35], [36], [37]]. Other studies had found that BCs with higher specific surface area (>400 °C) have greater surface meso- and microporosity [36,38,39], which caused by the loss of volatile matter [40,41].

In addition, the capabilty of biochar can act as a habit of microorganism growth should be added, which is very important for composting due to it is a biological-controlled process [[42], [43], [44]]. Different composting conditions influence significantly the community structure and metabolic intensity of composting microorganisms, leading to differences in the compost conversion efficiency [45]. Therefore, understanding the dynamics of microbial communities during composting is particularly important for regulating the composting process and improving compost quality [46].

Many existing researches on composting are conducted in pilot plants using the Rutgers static composting system, or classic windows composting system, or use industrial composting facilities [[47], [48], [49]]. In this research, we independently developed a simple composting simulation reactor (ZL 2021 2 1681205.2) with a collection device for ammonia and nitrous oxide supporting facilities. It uses common materials as raw materials, has low production cost, simple production process, high operability of the device, simple operation steps, and is convenient for real-time monitoring of the loss of nitrogen-containing gas during the composting process. By characterizing the changes in the microbial community structure as well as the nitrogen transformation (including nitrogen loss) and fractionation during composting, we elucidated the effect of biochar prepared at different pyrolysis temperatures on microbially driven conversion and retention of nitrogen. The study provided an important theoretical basis for the application of BC in improving the compost-related processes.

## 2 Materials and methods (see Appendix 1 for detailed introduction)

2.1 Materials and experimental set-up.

Raw materials for composting were sheep dung (D), mushroom cultivation residues (FR), rice husks, and rice husk biochar prepared at three pyrolysis temperatures. The experiment had a control group and three treatment groups, each in three replicates. B0: control group (feedstock material + rice husks); B1: B0 + biochar pyrolysed at 450 °C; B2: B0 + biochar pyrolysed at 550 °C. B3: B0 + biochar pyrolysed at 650 °C (Fig. S1). In addition, compost samples from 12 composting reactors were collected on the 6th, 21st and 42nd day after the start of composting, and labeled as M1 (mesophilic stage 1), T (thermophilic stage) and M2 (mesophilic stage 2), respectively. The properties of compost materials are shown in Table 1.

**Table 1:** Properties of composting materials.

Materials	Moisture content, %	pH_water_	TN, %	TC, %	C/N ratio	CEC, cmol kg^−1^	SSA, m^2^ g^−1^
Sheep dung	74.5 ± 1.1 a	7.50 ± 0.2 c	2.58 ± 0.06 a	41.2 ± 1.6 e	15.9 ± 1.3 e	–	–
Mushroom residue	41.9 ± 0.7 b	6.90 ± 0.1 c	0.98 ± 0.05 b	65.6 ± 0.8 a	66.9 ± 0.9 c	–	–
Rice husk	12.6 ± 0.5 c	7.20 ± 0.2 c	0.89 ± 0.02 bc	55.3 ± 1.1 bc	62.2 ± 1.1 d	–	–
Biochar pyrolysised at 450 °C	4.1 ± 0.2 d	8.70 ± 0.1 b	0.83 ± 0.05 cd	56.2 ± 0.5 b	67.7 ± 1.5 c	12.5 ± 1.5 a	39.2 ± 2.1 c
Biochar pyrolysised at 550 °C	3.9 ± 0.4 d	8.93 ± 0.2 ab	0.74 ± 0.06 de	52.9 ± 0.3 c	71.5 ± 1.1 b	10.8 ± 0.8 ab	43.7 ± 1.1 b
Biochar pyrolysised at 650 °C	3.0 ± 0.3 d	9.40 ± 0.4 a	0.62 ± 0.04 e	48.5 ± 0.8 d	78.2 ± 0.9 a	9.4 ± 0.8 b	53.5 ± 1.7 a

Note: TN: Total nitrogen. TC: Total carbon. CEC: Cation exchange capacity. SSA: Specific surface area.

### 2.1 Methods

#### 2.1.1 Basic indicators of compost and biochar microstructure

Three thermometers were positioned evenly on each reactor. The compost extract was used to obtain the pH and EC value of the compost using a multi-parameter water quality analyzer. The biochar microstructure was obtained by an ultra-high resolution field emission scanning electron microscope.

#### 2.1.2 Nitrogen and carbon in compost

The contents of total carbon (TC), total nitrogen (TN), ammonium nitrogen (NH_4_^+^-N) and nitrate nitrogen (NO_3_^−^-N) of the compost extract were obtained by using a continuous flow analyzer.

#### 2.1.3 Release of nitrogen-containing gases during composting

The ammonia collection device (ACD) was composed of an annular groove-shaped base and a transparent polymethyl methacrylate cylinder (Fig. S1). The nitrous oxide collection device (NCD) was composed of 1-cm-thick polyvinyl chloride (PVC) plastic sheets welded together to make a square-shaped grooved base and a PVC box (Fig. S1). The sampling frequency was once every three days (about 9:30 a.m.) starting from the day of composting.

The nitrogen loss rate (NLR) was calculated on the premise that the absolute amount of ash content during the composting process was unchanged:

NLR (%) = (N_1_–H_1_/H_43_ × N_43_)/N_1_ × 100%, whereby N_1_ and N_43_ were the mass fractions of total nitrogen on the 1st and 43rd day of composting, respectively, and H_1_ and H_43_ were the mass fractions of the ash on the 1st and 43rd day of composting, respectively. The mass fractions were based on dry matter (%) [46,50].

#### 2.1.4 Microbial community structure

All the collected samples were transferred to the sequencing company in liquid nitrogen for analysis of genes related to carbon- (*cbbL a*nd *cbbM*) and nitrogen-fixation (*nifH*). Besides, we conducted a statistical analysis of bioinformatics for OTUs at a similar level of 97%.

#### 2.1.5 Statistics

Statistical analyzes were performed using Microsoft Excel 2016, SPSS Statistics 20.0 (IBM, Armonk, New York, USA) and R package vegan (v2.5-5, v2.5-6, v1.0.12). QIIME (v.1.8.0) for alpha diversity analysis (chao1, shannon). Adobe Illustrator CC 2017, GraphPad Prism (v8.0.1) and R packages (pheatmap v1.0.12, ggplot2 v3.2.1, ggord v1.15, and corrplot v0.84) were used to draw figures. In addition, FAPROTAX (v1.2.2) was used for the microbial community function prediction.

## 3 Availability of data and materials

The complete sequencing data sets have been deposited in the NCBI Sequence Read Archive (SRA) database under the accession number PRJNA688812 (*cbbL*), PRJNA68862 (*cbbM*) and PRJNA688639 (*nifH*).

## 4 Results

### 4.1 Basic indicators of biochar and the composting system

#### 4.1.1 Basic indicators and structure of biochar (see Appendix 2)

##### 4.1.1.1 Temperature, pH and total carbon of compost heap

The composting lasted for 42 days. The average temperature of the compost in the initial stage of composting (stage M1) was 44.6 °C–47.0 °C, and then the temperature increased rapidly (51.9 °C–63.9 °C) into the high-temperature period of composting (stage T) (Fig. S3). After entering the stable period (stage M2), the compost temperature (52.7 °C–61.0 °C in B0, 47.1 °C–56.2 °C in B1, B2 and B3) gradually dropped to slightly above the ambient temperature (34.5 °C ± 2.44 °C) and remained relatively stable. In addition, the heap temperature of the B0 group (control treatment without biochar) rose to above 50 °C on the 6th days and above 60 °C on the12^th^ day of composting. The heap temperature of the three treatments supplemented with biochar increased to 60 °C for 4 days earlier than that of B0 treatment (12th day) (Fig. S3).

There was no significant difference among the four groups in pH during the composting process. The pH increased from 7.10 to 7.32 in the M1 stage to 8.65–8.96 in the M2 stage (*p* ≤ 0.05) (Fig. S3).

Compared with the stage M1, the total carbon concentration (TC) of B0, B1, B2 and B3 decreased by 11.1%, 8.40%, 7.98% and 6.56% respectively at the end of composting, while they were 558 ± 26.4 g/kg, 611 ± 15.3 g/kg, 631 ± 19.3 g/kg, and 626 ± 6.20 g/kg at stage M1. (Fig. S3).

### 4.2 Nitrogen in compost

#### 4.2.1 Total, kjeldahl, ammonium, and nitrate nitrogen

**Fig. 1:**
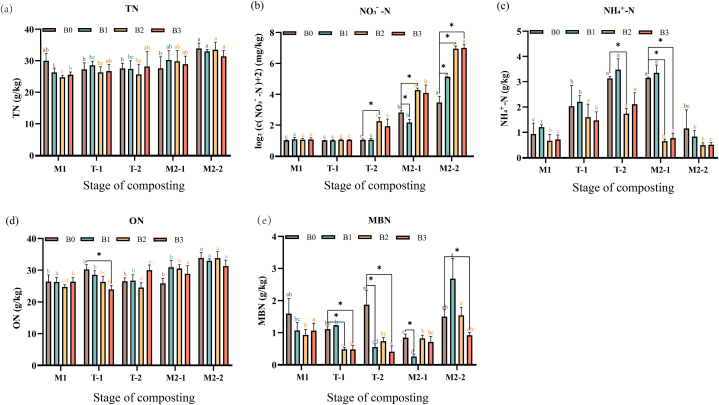
Biochar-induced changes in nitrogen forms during composting. Note: M1: mesophilic stage 1. T-1: the first half of the thermophilic stage. T-2: the second half of the thermophilic stage. M2-1: the first half of the stable stage. M2-2: the second half of the stable stage. B0: control group (feedstock material + rice husks); B1: B0 + biochar pyrolysed at 450 °C; B2: B0 + biochar pyrolysed at 550 °C. B3: B0 + biochar pyrolysed at 650 °C. The data are means +standard error (n = 3). Different lower case letters denote significant differences among different stages in the same treatment (*p* ≤ 0.05). *indicates the significant differences between the three biochar treatments (B1–B3) and the control B0 in the same period (*p* ≤ 0.05).

Compared with the stage M1 (45.6 ± 1.08 °C), the total nitrogen concentration (TN) increased by 13.2% (B0), 25.6% (B1), 35.5% (B2) and 22.8% (B3) respectively at the end of composting (stage M2, 53.2 ± 4.54 °C) (Fig. 1a). There was no difference in TN among the four treatments at different stages, but there was a significant increase in each treatment between the commencement and completion of composting (*p* ≤ 0.05).

The concentration of nitrate nitrogen (NO_3_^−^-N) in the four treatments increased by orders of magnitude from 0.08 ± 0.04 mg/kg in stage M1 to 9.38 ± 3.22 mg/kg (B0), 32.8 ± 0.16 mg/kg (B1), 122 ± 15.3 mg/kg (B2), and 128 ± 22.6 mg/kg (B3) (*p* ≤ 0.05) (Fig. 1b). The dynamics of ammonium nitrogen (NH_4_^+^-N) (Fig. 1c) differed from that of TN. Although NH_4_^+^-N in the four treatments did not differ between the M1 and the M2-2 stages (on average 0.81 ± 0.27 g/kg), there was a significant increase in stage T (58.3 ± 4.72 °C), reaching 3.28 ± 0.17 g/kg (average of B0 and B1) and 1.73 ± 0.28 g/kg (average of B2 and B3).

#### 4.2.2 Organic and microbial biomass nitrogen and nitrogen loss rate

The organic nitrogen concentration (ON) of the four treatments was in the range of 23.7–26.8 g/kg in stage M1 (Fig. 1d). By the stage M2-2, the concentration of ON in the four groups was 32.9 ± 8.8 g/kg, having increased by 24.0%–36.9%. Similarly to TN, there was almost no difference in ON between the four treatments at different stages (except for T), but there was a significant increase in each treatment during the composting process (*p* ≤ 0.05).

The microbial biomass nitrogen (MBN) showed substantial fluctuations throughout the composting process (Fig. 1e): 0.84–1.59 g/kg (B0), 0.26–2.68 g/kg (B1), 0.48–1.54 g/kg (B2), and 0.48–1.06 g/kg (B3). There was no significant difference in MBN of B0 and B3 at the beginning and the end of composting, whereas MBN of B1 and B2 was significantly higher at the end compared with the beginning of composting. At the end of composting, MBN was significantly higher in B1 than the other three treatments (*p* ≤ 0.05) (Fig. 1e).

According to the formula, the nitrogen loss rates (NLR, %) of the four treatments were 31.4 ± 2.73 (B3) < 39.8 ± 3.72 (B1) = 43.6 ± 1.56 (B2) < 54.5 ± 3.34 (B0) (*p* ≤ 0.05) (Fig. S3).

#### 4.2.3 Ammonia and nitrous oxide emissions

**Fig. 2:**
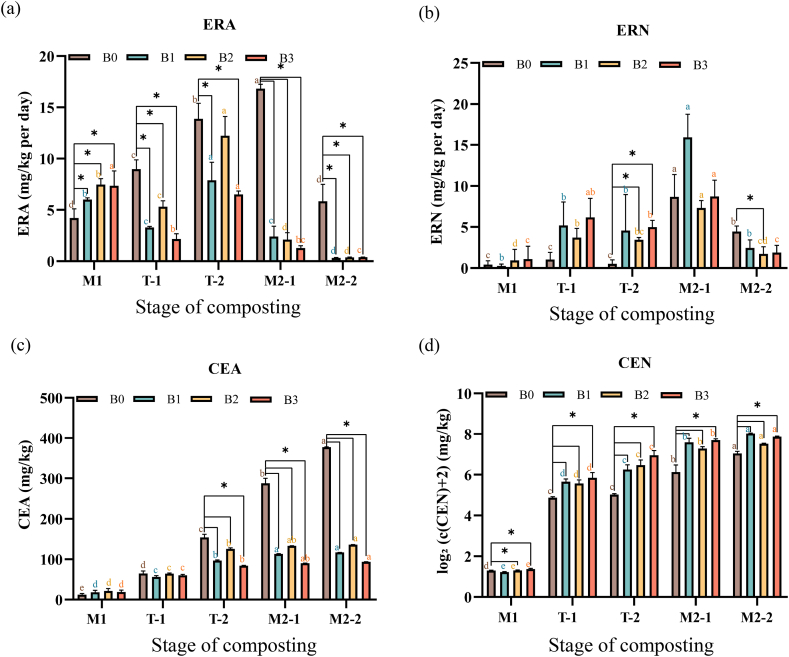
Biochar-induced changes in nitrogen forms during compostingNote: M1: mesophilic stage 1. T-1: the first half of the thermophilic stage. T-2: the second half of the thermophilic stage. M2-1: the first half of the stable stage. M2-2: the second half of the stable stage. B0: control group (feedstock material + rice husks); B1: B0 + biochar pyrolysed at 450 °C; B2: B0 + biochar pyrolysed at 550 °C. B3: B0 + biochar pyrolysed at 650 °C. The data are means +standard error (n = 3). Different lower case letters denote significant differences among different stages in the same treatment (*p* ≤ 0.05). *indicates the significant differences between the three biochar treatments (B1–B3) and the control B0 in the same period (*p* ≤ 0.05).

During the composting process, both the ammonia emission rate (ERA) and the nitrous oxide emission rate (ERN) generally changed along with the alteration of the heap temperature, i.e. they showed a trend of first increasing and then decreasing. The average ERA of B0 (range 4.22–16.8 mg kg^−1^ d^−1^) was greater than that of the other three treatments (range 0.32–12.2 mg kg^−1^ d^−1^) in the stages from T-1 (54.1 ± 1.77 °C) onwards (but the difference with respect to B2 was not significant in the T-2 stage (62.5 ± 1.19 °C)) (Fig. 2a).

The maximum ERN of the all four treatments was in the stage M2-1 (56.6 ± 3.06 °C) (Fig. 2b). The maximum ERN of B0, B2 and B3 ranged between 6.37 and 11.8 mg/kg/d, and it was 15.9 ± 2.81 mg kg^−1^ d^−1^ in B1.

By the end of composting (stage M2-2, 49.7 ± 2.58 °C), the cumulative amounts of NH_3_ emission (CEA, mg·kg^−1^) in the four treatments were 93.6 ± 0.26 (B3) <117 ± 0.21 (B1) <136 ± 0.22 (B2) <377 ± 0.89 (B0) (*p* ≤ 0.05), i.e. the amount of ammonia released by B0 was 3.2 times, 2.8 times and 4.0 times that of B1, B2 and B3, respectively (Fig. 2c).

In contrast to ammonia emission, the cumulative amount of nitrous oxide emitted (CEN) was significantly lower in B0 than B1, B2 and B3 throughout the composting process (Fig. 2d). By the end of composting, the CEN (mg·kg^−1^) of the four treatment groups were 130 ± 8.7 (B0) <181 ± 3.3 (B2) <233 ± 3.5 (B3) <250 ± 4.4 (B1) (*p* ≤ 0.05), i.e. the amount of nitrous oxide released by B1, B2 and B3 was, respectively, 2.0 times, 1.4 times and 1.8 times that of B0 (Fig. 2d).

### 4.3 Microbial community structure

#### 4.3.1 OTU distribution and taxonomic analysis

**Fig. 3:**
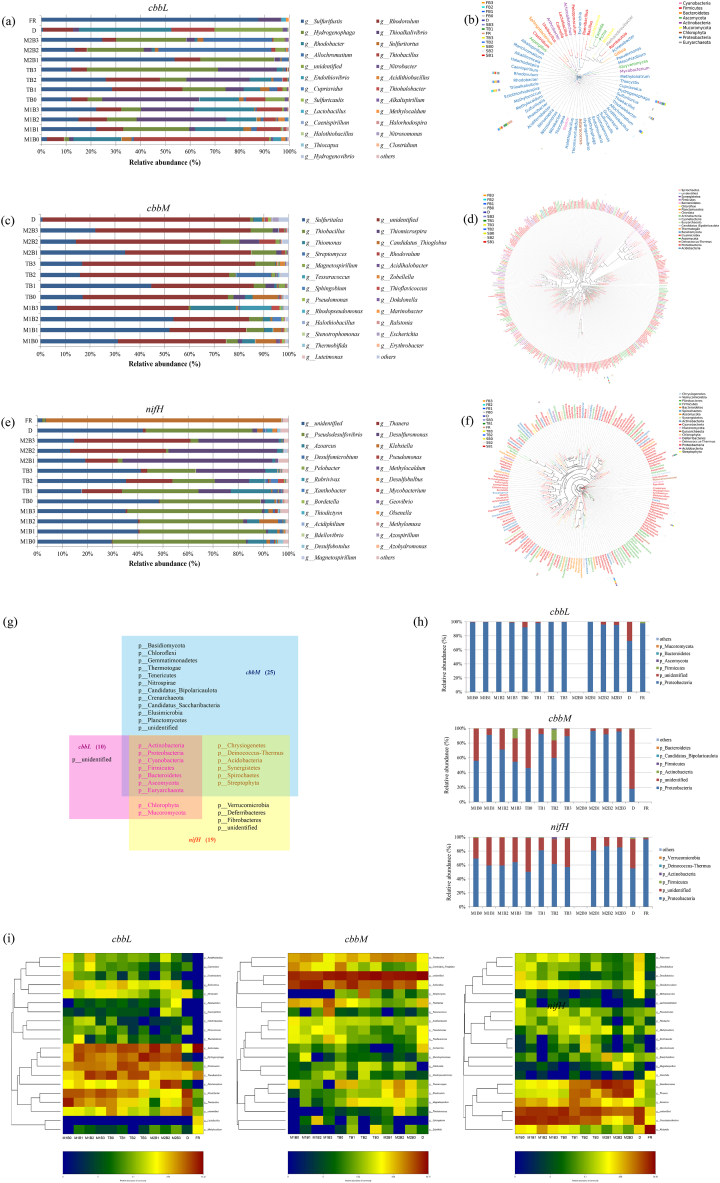
Taxonomic analysis and abundance of different microorganisms in various biochar treatments during the composting process.

In this study, we detected a total of 800, 6112 and 1089 OTUs containing *cbbL*, *cbbM* and *nifH,* respectively (Table S1). In stage M1, the three *cbbL*-containing genera with the highest relative abundance were *Thiobacillus* (54%), *Rhodobacter* (19%) and *Rhodovulum* (6.9%) in B0, whereas the dominant genera in B1, B2 and B3 were *Sulfurifustis* (15%–23%), *Rhodovulum* (10%–12%), *Hydrogenophaga* (8%–27%), *Thioalkalivibrio* (2.5% in FB1, 36% in FB2, 21% in FB3), *Rhodobacter* (12%–25%), and *Thiobacillus* (4.8%–11%) (Fig. 3a). The sum of the relative abundance of these six genera was greater than 91% in all samples regardless of the treatment and composting duration.

After entering the T stage, the dominant *cbbL*-containing genera in the all four treatments were *Sulfurifustis* (12%–29%), *Rhodovulum* (6.1%–28%), *Hydrogenophaga* (53% in SB3, but 5.8%–18% in SB0, SB1 and SB2), *Thioalkalivibrio* (7.4% in SB1, but 23%–40% in SB0, SB2 and SB3) and *Rhodobacter* (16% in SB0, but 2.5%–8.9% in SB1, SB2 and SB3) (Fig. 3a). In the stable (T) period, the most abundant genera in TB1 were *Sulfurifustis* (54%), *Rhodovulum* (10%) and *Hydrogenophaga* (30%), whereas the dominant genera in TB2 and TB3 were *Sulfurifustis* (13% and 25%, respectively), *Hydrogenophaga* (23% and 28%) and *Allochromatium* (46% and 28%) (Fig. 3a).

Combining the heatmap and the genus-level phylogenetic tree based on the representative sequences of the most abundant OTUs, we found that the five *cbbL*-containing genera with the highest relative abundances in all samples were *Sulfurifustis > Hydrogenophaga > Thioalkalivibrio > Rhodobacter > Rhodovulum* (Fig. 3b, i).

Although 385 genera containing *cbbM* were detected (323 more than those containing *cbbL*) (Table S1), the dominant genera were distinct from those revealed by *cbbL* sequencing. The sum of the relative abundances of *Sulfuritalea* and *unidentified*, which had the highest relative abundances in most samples, was only 60% in FB3, and 75%–86% in the remaining samples. In addition, other *cbbM*-containing genera with relatively high abundance were *Candidatus_Thioglobus* in FB0 (8.5%) and SB0 (10%), and *Thiomonas* in FB3 (18%). In the stable (M2) stage of composting, *Sulfuritalea*, *unidentified* and *Thiobacillus* were the dominant genera in B1, B2 and B3, respectively, with relative abundances of 10%, 7.6% and 9.0% (Fig. 3c).

Combining the heatmap and the genus-level phylogenetic tree, we found that the order of relative abundance of *cbbM*-containing genera in most samples was *Sulfuritalea* > *Thiobacillus* > *Thiomonas > Candidatus_Thioglobus > Thiomicrospira > Streptomyces > Tessaracoccus* > *Rhodovulum*. The first five genera and *Rhodovulum* are belong to *Proteobacteria*, and the other two belong to *Actinobacteria* (Fig. 3d, i).

By analyzing the community composition of nitrogen-fixing microorganisms containing *nifH*, we found that the dominant genera in the four treatments in stage M1 was *Pseudodesulfovibrio* (41%–54%). The dominant genera in B0 did not change during the composting process. However, after entering stage T, the dominant *nifH*-containing genera of the other three treatments were *Pseudodesulfovibrio* (17%–31%), *Desulfuromonas* (12%–28%), *Thauera* (2.5%–19%) and *Azoarcus* (4.7%–16%), and then changed to *Desulfuromonas* (32%–60%) and *Thauera* (13%–47%) in stage M2 (Fig. 3e).

We found that the *nifH*-containing genera with relatively large relative abundance in most samples were *Desulfuromonas > Thauera > Klebsiella > Azoarcus > Pseudodesulfovibrio > Desulfomicrobium > Bradyrhizobium > Rubrivivax > Desulfobotulus > Methylocaldum,* all belong to *Proteobacteria* (Fig. 3f, i). *Proteobacteria* was the most abundant phylum among the three functional types of microorganisms. *Actinobacteria, Proteobacteria, Cyanobacteria, Firmicutes* and *Bacteroidetes* were the bacterial phyla in all three functional types of microorganisms (containing *cbbL*, *cbbM* and *nifH*), and they shared *Euryarchaeota* (archaeal) and *Ascomycota* (fungal) phyla (Fig. 3g and h).

#### 4.3.2 Alpha and beta diversity analysis

**Fig. 4:**
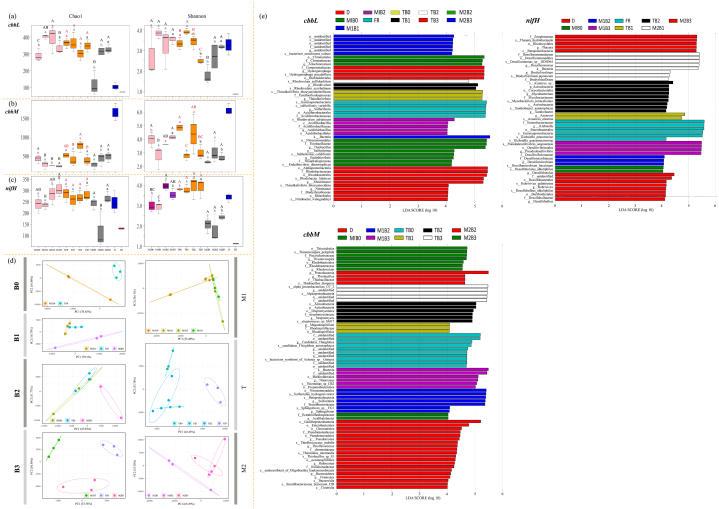
Diversity analysis and Linear discriminant analysis effect size (LEfSe) analysis of different functional types of microorganisms in various treatments during the composting process. Note: M1: mesophilic stage 1. T: thermophilic stage. M2: stable stage (mesophilic stage 2). B0: control group (feedstock material + rice husks); B1: B0 + biochar pyrolysed at 450 °C; B2: B0 + biochar pyrolysed at 550 °C. B3: B0 + biochar pyrolysed at 650 °C. Different lower case letters denote significant differences among different stages in the same treatment (*p* ≤ 0.05). Different uppercase letters denote significant differences among different treatment in the same stages (*p* ≤ 0.05). No OTUs containing any of the three functional genes were recorded in TB0. (a, b, c) Alpha diversity indices of *cbbL*, *cbbM* and *nifH*. (d) Beta diversity analysis (principal component analysis): The distribution of samples from different treatments in various composting stages. (e) LEfSe analysis of *cbbL*, *cbbM* and *nifH*. LDA distribution histograms were ranked according to the effect size (*p*-value<0.05, LDA>3).

As shown by the alpha diversity indices, the diversity index (Shannon) and the richness index (Chao1) of the *cbbL*-containing OTUs in the four treatments showed similar treatment effects. The indices of B1 and B2 decreased significantly after composting entered the stable period (F > T), whereas those of B3 did not change significantly during composting. Finally, for both indices, TB1 was lower than TB2 and TB3 (*p* ≤ 0.05) (Fig. 4a).

Regarding the *cbbM*-containing OTUs, there was no significant change in alpha diversity indices among the four treatments during the entire composting process. At the end of composting, there was no significant difference in the two indices in the four treatments (Fig. 4b).

As for the *nifH*-containing OTUs, the Chao1 index of B0, B1 and B3 did not change significantly throughout composting, whereas that of B2 decreased significantly after the compost entered stage M2 (B2 lower than B0, B1and B3, *p* ≤ 0.05). In contrast, Shannon index showed the order B0 = B1<B2 = B3 (*p* ≤ 0.05) in the initial (M1) stage, and was not different among the treatments in the high-temperature period (stage T). During the composting process, there was a gradual decrease between the T and M2 stages, with the treatment order B0<B2<B1 = B3 (*p* ≤ 0.05) (Fig. 4c).

Considering the PCA diagrams of the three databases (*cbbM*, *cbbL* and *nifH*), the microbial community composition of the four treatments changed significantly over time, in particular, the community composition of the four treatments in the M2 stage is significantly different from that of the M1 and T stages. In the M1 stage, the microbial community composition among the four treatments was somewhat similar, especially that of B2 and B3. Subsequently, the differences between the four treatments gradually increased. At the M2 stage, and the community composition of B2 and B3 was similar, and both were significantly different from B1 (Fig. 4d).

#### 4.3.3 Multivariate statistical analysis and prediction of gene functions

The results of ANOSIM analysis showed there was a medium difference (R-value 0.703, 0.751) in the composition of *cbbL*- and *cbbM*-containing OTUs among the treatments, and a big difference (R-value 0.856) in *nifH*-containing OTUs (Appendix 3).

**Fig. 5:**
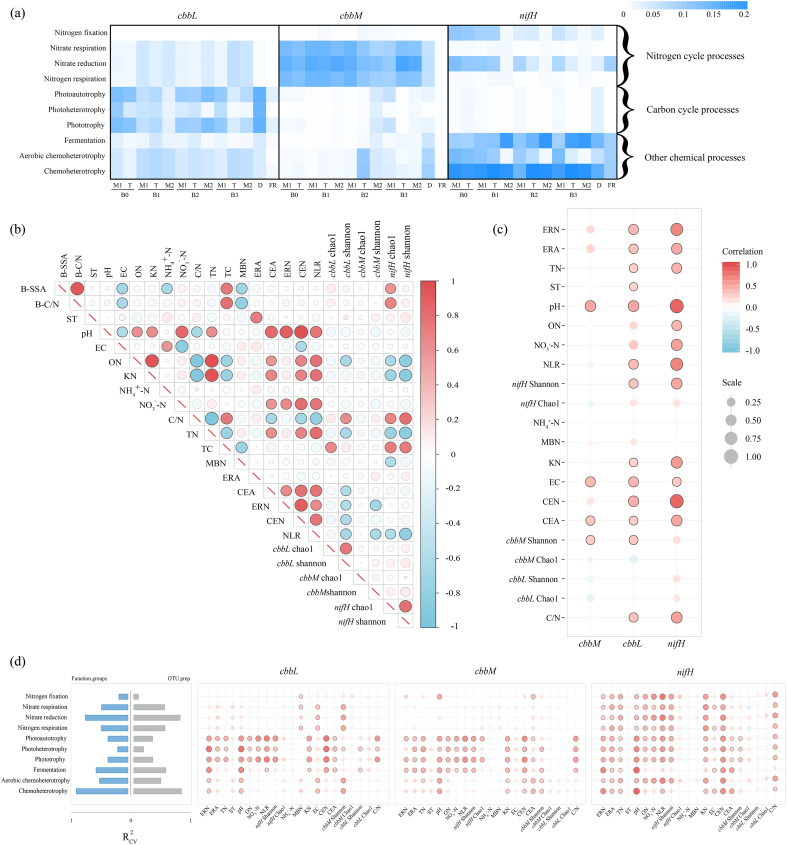
Prediction of gene function based on OTUs and correlation analysis between indicators.

Using the Kruskal-Wallis test, the differential OTUs at the genus level (*p*-value≤0.05) were selected. There were 27 differential OTUs containing *cbbL*. Combined with linear discriminant analysis effect size (LEfSe) analysis (LDA>3), we filtered OTUs with significantly different abundance among the groups (i.e., biological indicators), and ordered them in the descending order of *p*-values (Top 10): *Rhodovulum, Nitrobacter, Allochromatium, Sulfurifustis, Endothiovibrio, Thiobacillus, Acidithiobacillus, Sulfuritortus, Hydrogenophaga* and *Thioalkalivibrio* (Fig. 4e). In addition, there were 103 differential OTUs containing *cbbM* in the descending order of *p*-values (Top 10): *Halomonas, Thiomicrospira, Acidihalobacter, Sphingobium, Pseudomonas, Thiobacillus, Rhodovulum, Thioflavicoccus, Candidatus_Thioglobus* and *Magnetospirillum* (Fig. 4e). Furthermore, there were 42 differential OTUs containing *nifH* in the descending order of *p*-values (Top 10): *Rubrivivax, Bradyrhizobium, Mycobacterium, Desulfobulbus, Klebsiella, Desulfomicrobium, Azoarcus, Thauera, Desulfuromonas* and *Pseudodesulfovibrio* (Fig. 5e).

We also predicted the main functions of the microbial community from OTUs. The top 10 functions with the highest annotation volume were nitrogen fixation, nitrate respiration, nitrate reduction, nitrogen respiration, photoautotrophy, photoheterotrophy, phototrophy, fermentation, aerobic chemoheterotrophy and chemoheterotrophy (Fig. 5a).

The abundance of gene functions predicted by the OTUs in the three databases (*cbbL, cbbM* and *nifH*) differed across samples. In the OTUs containing *cbbL*, the abundance of functions related to the carbon cycle was relatively high, such as photoautotrophy, phototrophy and photoheterotrophy. However, in the OTUs containing *cbbM*, the abundance of functions related to the nitrogen cycle was higher than those related to the other cycles. In the OTUs containing *nifH*, the five most abundant functions were chemoheterotrophy, fermentation, aerobic chemoheterotrophy, nitrate reduction and nitrogen fixation (Fig. 5a).

### 4.4 Correlations between the compost physicochemical properties and microbial community

We analyzed the correlation between the physicochemical properties of compost and the diversity of microbial community (Fig. 5b). ERA was and positively correlated to ST (*p* ≤ *0.05*). Whereas ERN, CEA, CEN, and NLR were respectively positively correlated with compost pH or NO_3_^−^-N. In addition, ON, TN and NO_3_^−^-N had a significant positive correlation with pH (*p* ≤ *0.05*).

Shannon index and Chao1 index of *nifH* had a significant negative correlation with ON or TN (*p* ≤ *0.05*). ON, TN, ERA, ERN, CEN and NLR had a significant negative correlation with Shannon index of *cbbL*. Moreover, NLR had a significant negative correlation with Shannon index and Chao1 index of *nifH* or Shannon index of *cbbM* (*p* ≤ *0.05*) (Fig. 5b).

Through an in-depth analysis of the correlation between the physicochemical properties of the compost heap and the expression level of OTUs in the three databases (*cbbL, cbbM* and *nifH*), we found a number of positive correlations. In particular, the expression levels of OTUs containing *cbbL* and *nifH* were positive correlated with ERN, ERA, TN, pH, NLR, Shannon index of *nifH*, EC, CEN, CEA and C/N (*p* ≤ *0.05*) (Fig. 5c and d). In addition, from the standardized histograms, we determined the ratio of the top 10 gene functions predicted by OTUs in this study (i.e., the proportion of one type of gene function to the total gene function). The five functions with the highest proportions were chemoheterotrophy, nitrate reduction, fermentation, aerobic chemoheterotrophy and nitrogen respiration (Fig. 5d).

## 5 Discussion

### 5.1 The effect of biochar prepared at different pyrolysis temperatures on composting (see Appendix 2)

#### 5.1.1 The changes in the microbial community structure as well as the nitrogen transformation and fractionation during composting

Microorganisms play a special role in the energy flow and material circulation in the ecosystem [51]. Different microbial functional communities jointly regulate and drive the biogeochemical cycling [[52], [53], [54]]. The organic matter in the composting mixture underwent microbially mediated mineralization and humification, and the energy generated promoted composting and increased the temperature of the compost heap. We found *Bacillus*, which could decompose organic substances, including organic sulfur compounds, organic nitrogen, etc. in this research. There was also *Pseudomonas*, with a strong capacity to decompose organic matter [55]. Other genera with high capacity to decompose lignin and cellulose [56] were found, such as *Brevibacterium, Butyrivibrio, Cellulomonas, Cellvibrio, Clostridium, Micromonospora, Nocardia, Thermobifida, Pseudoalteromonas, Ruminococcus, Streptomyces, Thermomyces*, etc. (Table S2).

The change in temperature in turn affected the microbial community structure and then the decomposition rate of organic matter [57,58]. In this study, the heap temperature of the B0 treatment (no biochar control) rose to above 50 °C after 6 d and above 60 °C after 12 d of composting. The addition of biochar shortened the heating time by nearly half (Fig. S3), indicating that biochar significantly improved the efficiency of composting. That was, as an auxiliary composting material, biochar accelerated decomposition of organic matter.

The heat accumulated through decomposition rapidly increased the temperature of the compost heap [59], and the microbial community structure also changing significantly. Heat-resistant or thermophilic microbes gradually became dominant [60,61]. Subsequently, the compost heap temperature gradually dropped to slightly above the ambient temperature (Fig. S3), and heat-labile microbes regained dominance. However, the proliferation of microbes can also lead to competitive inhibition [45].

The pyrolysis temperature was found to have a strong correlation with changes in the structure and physical and chemical properties of biochar, such as micropores, which provide a safe habitat for microorganisms [42]. Carbon and nitrogen biofixation processes are critical in biogeochemical cycles [[62], [63], [64]]. We explored whether the addition of biochar could improve the fixation of carbon and nitrogen in the entire system by changing the diversity of autotrophic microorganisms and microorganisms with nitrogen fixation function. In this study, the diversity and richness of the three functional types of microbes showed a downward trend in abundance during the composting process. At the end of composting, the community composition of B2 and B3 was similar, but significantly different from that of B1 (Fig. 3).

Compared with the stage M1, the reduction rate of the total carbon concentration (TC) of the compost heap is B0>B1>B2>B3, which means that the higher the carbonization temperature of biochar, the less the loss of carbon in the compost. This might be due to the adsorption effect of the microporous (Fig. 5) of the biochar or the increased in the abundance of autotrophic microorganisms. Among the *cbbL*-containing and the *cbbM*-containing biological indicators, *Rhodobacter* was one of the dominant genera in all treatments in stage M1, had extensive metabolic capabilities, and they were deeply involved in the biogeochemical cycling of sulfur and carbon [[65], [66], [67]]. *Sulfuritalea, Hydrogenophaga, Thiobacillus* and *Candidatus_Thioglobus* were the dominant genera in treatments with biochar (B1, B2 and B3), while *Thiomonas* was one of the dominant genera in B3. In *Hydrogenophaga*, two species (*H. pseudoflava* and *H. taeniospiralis*) were capable of anaerobic nitrate respiration and had the function of denitrification [37]. *Thiobacillus, Thiomonas* and *Sulfuritalea* are ammonia-oxidizing bacteria, and *Sulfuritalea* could participate in denitrification [68,69]. In addition, studies have shown that *Candidatus_Thioglobus* plays a key role in carbon fixation and denitrification [[70], [71], [72]]. This explained why OTUs associated with nitrogen cycle also showed abundance of microorganisms containing *cbbL* and *cbbM*.

Numerous microbial taxa are involved in the nitrogen cycle. Generally, 25 °C was the most suitable temperature for the growth of nitrifying bacteria, and their activity dropped sharply above 42 °C [73]. Some species in the genera *Pseudomonas* (B3, B2>B1, B0)*, Bacillus*, *Clostridium* (B3>B1, B2, B0) and *Micrococcus* also participate in ammonification, and the fungi with strong capacity to decompose nitrogenous organic compounds include many species in genera *Rhizopus* (B2, B0>B1, B3) and *Aspergillus* (B2, B1, B0 >B3).

Nitrogen underwent a series of biochemical reactions to be transformed into various forms during the composting process. However, the concentrations of total nitrogen (TN) and organic nitrogen (ON) were not significantly different among the four treatments (Fig. 1). Ammonification occurred mainly in the thermophilic (T) stage of composting. The complex organic nitrogen compounds in the composting mixture were converted to NH_4_^+^-N by microorganisms, and part of it was emitted in the form of NH_3_ or converted to NO_3_^−^-N through nitrification. A part of NH_4_^+^-N and NO_3_^−^-N was also assimilated into MBN by microorganisms. The majority of plant-available nitrogen is in the inorganic forms NH₄⁺ and NO₃⁻ (=mineral nitrogen).

Nitrification is a two-step process, ammonia-oxidizing bacteria (nitrite bacteria) first oxidize NH₄⁺ to NO₂⁻. This type of bacteria included *Nitrosomonas, Nitrosospira* and *Nitrosococcus* documented in the present study. Among them, the genus *Nitrosomonas* played a dominant role, and the most common species was *Nitrosomonas europaea*. Then, nitrate bacteria that oxidize NO₂⁻ to NO₃⁻ include *Nitrospira, Nitrococcus* and *Nitrocystis*. There were two main genera (*Nitrobacter* and *Nitrospira*) of nitrate bacteria found in this study (B2, B3 > B1, B0) (Table S3). Among them, the genus *Nitrobacter* was the main one, and the common species were *N. winogradskyi* and *N. agilis* [74]. This process requires a well-aerated environment [[75], [76], [77]]. Studies have shown that high concentrations of NH_4_^+^ (≥400 mg/kg) could inhibit nitrification and likely to cause the accumulation of NO_2_^−^; too high concentration of the final product NO_3_^−^ would also inhibit the activity of nitrite and nitrate bacteria [78,79].

In the present study, although the concentration of NH_4_^+^-N in the four treatments did not differ significantly between the M1 stage and the M2 stage of composting, there was a significant increase in the T stage. The concentration of NO_3_^−^-N increased sharply from the beginning of composting (0.08 ± 0.04 mg/kg) to B0 (9.38 ± 3.22) < B1 (32.8 ± 0.16) < B2 (122 ± 15.3) = B3 (128 ± 22.6) (p ≤ 0.05) later, accompanied by a decrease in the NH_4_^+^-N concentration (Fig. 1). The high concentration of NH_4_^+^-N in the B0 treatment likely inhibited the formation of NO_3_^−^-N.

Besides, nitrification is dependent on temperature. The most suitable temperature for autotrophic nitrification was 25–35 °C, but heterotrophic nitrification could be carried out at temperatures greater than 40 °C or even at 50–60 °C [80,81]. Thus, the nitrification reaction in compost was mainly heterotrophic. In addition, pH was also an important factor affecting nitrification. The optimal pH for nitrite and nitrate bacteria was 7–9 [82,83], and the pH of compost in the present study (7.10–8.96) was within that range (Fig. S3). Therefore, pH had little effect on nitrification in this study.

Denitrification occurred when the compost was poorly ventilated, facilitating emission of gaseous nitrogen such as nitrous oxide (N_2_O), nitric oxide (NO) and nitrogen dioxide (NO_2_), which impoverished compost as a source of nitrogen to be used in plant production [84,85]. During the composting process, both the ammonia emission rate (ERA) and the nitrous oxide emission rate (ERN) generally showed a trend of first increasing and then decreasing; the cumulative amount of N_2_O emission (CEN) in the B0 treatment was always significantly lower than that in the B1, B2 and B3 treatments. By the end of composting, the CEN was in the order B0<B2<B3<B1 (*p* ≤ 0.05) (Fig. 2). These findings suggested that the addition of biochar does not have a positive effect on reducing greenhouse gas emissions.

Denitrifying bacterial genera such as *Pseudomonas, Alcaligenes, Paracoccus, Bacillus, Citrobacter, Mesorhizobium, Thiobacillus* and *Rhodococcus* in this study (Table S2), carried out aerobic respiration under aerobic conditions, but reduced NO_3_^−^-N to NH_3_ and N_2_ under anoxic conditions, which was an important reason for nitrogen loss during composting [85,86]. Among the *nifH*-containing biological indicators (at the genus level) (Fig. 5), *Thauera* may use oxygen and nitrogen oxides as electron acceptors and alter the metabolic state between aerobic respiration and denitrification [46,87,88]. The above findings explained, at least partly, the OTU expression level of *nifH* being significantly and positively correlated with CEA, CEN and TN, and the five most abundant functions being chemoheterotrophy, fermentation, nitrate reduction, nitrogen fixation and aerobic chemoheterotrophy in the OTUs containing *nifH*.

The amount of ammonia emitted (CEA) in the B0 treatment (377 ± 0.89 mg kg^−1^) was 3.2 times, 2.8 times and 4.0 times that in B1, B2 and B3, respectively (Fig. 2). This result indicated that the addition of biochar significantly reduced the loss of nitrogen in the form of NH_3_ from the composting system, which might have been due to the adsorption of NH_3_ by the rich microporous structure of biochar. Studies have shown that *Pseudomonas aeruginosa, Bacillus subtilis and Bacillus licheniformis* isolated from compost exhibited multiple beneficial traits including nitrogen fixation and phosphorus solubilisation, etc., and identified that plants treated with *P. aeruginosa* had a nitrogen content of 40.6% higher than that of the untreated controls, which further confirmed the nitrogen fixation function of *P. aeruginosa* [[89], [90], [91]].

Recently, biochar amendment has been considered to be an efficient and promising technology to adsorb greenhouse gas (GHG), ammonia and extractable ammonia [[92], [93], [94]] and increases the organic matter degradation [18,95]. In this study, the nitrogen loss rate (NLR) in the four treatments was B3<B1=B2<B0 (Fig. S3), which showed that biochar decreased markedly the nitrogen loss during composting. Results clearly indicated that the biochar significantly reduced the nitrogen loss that could be due to biochar adsorbing urea and uric acid, enhancing the adsorption of gaseous NH_3_ as well as improving the microbial growth and composting efficiency [96,97]. In addition, biochar not only improved the compost structure and permeability, but also provided living space for microbes, thus promoting the degradation of organic matter and increasing soil fertility [16,[98], [99], [100]].

## 6 Conclusions

Under the interaction of biochar and compost material, the decomposition of organic matter was accelerated, while reduced the loss of nitrogen and carbon, and the loss from small to large was B3 (31.4 ± 2.73)< B1 (39.8 ± 3.72) = B2 (43.6 ± 1.56) < B0 (54.5 ± 3.34). Denitrifying bacterial genera such as *Pseudomonas, Alcaligenes, Paracoccus, Bacillus, Citrobacter, Mesorhizobium, Thiobacillus* and *Rhodococcus* in this study was an important reason for nitrogen loss during composting. The abundance of autotrophic microorganisms (*Sulfuritalea, Hydrogenophaga, Thiobacillus, Thiomonas* and *Candidatus_Thioglobus, etc.*) in treatments with biochar (B1, B2 and B3) were higher than that in B0. At the end of composting, the community composition of B2 and B3 was similar, but significantly different from that of B1 and B0. Moreover, most microorganisms were involved in chemoheterotrophy, nitrate reduction, fermentation, aerobic chemoheterotrophy and nitrogen respiration during the composting process. This lays a theoretical foundation for the application of biochar in composting to enhance compost-related processes.

## Declarations

### Author contribution statement

Haihou Wang, Tianyun Shao, Xiaohua Long: Conceived and designed the experiments; Performed the experiments; Wrote the paper. Tianyun Shao, Zed Rengel: Analyzed and interpreted the data; Wrote the paper. Tianyun Shao, Yujie Zhou: Contributed reagents, materials, analysis tools or data.

### Data availability statement

The data that has been used is confidential.

### Additional information

Supplementary content related to this article has been published online at [URL].

Nitrogen forms measured were: total (TN) (a), nitrate (NO_3_^−^-N) (b), ammonium (NH_4_^+^-N) (c), organic (ON) (d), and microbial biomass nitrogen (MBN) (e).

Nitrogen emissions include emission rates of ammonia (ERA) (a) and nitrous oxide (ERN) (b), and cumulative amounts of emitted ammonia (CEA) (c) and nitrous oxide (CEN) (d).

Note: M1: mesophilic stage 1. T: thermophilic stage. M2: stable stage (mesophilic stage 2). B0: no biochar control (feedstock material + rice husks); B1: B0 + biochar pyrolysed at 450 °C; B2: B0 + biochar pyrolysed at 550 °C; B3: B0 + biochar pyrolysed at 650 °C; D: sheep dung; FR: mushroom cultivation residues. The data are means (n = 3). No OTUs containing any of the three functional genes were recorded in TB0. (a, c, e) Histograms of microbial composition at the genus level. (b, d, f) Genus-level evolutionary trees. The representative sequences of the OTU of the most abundant genera were selected to build the tree; the outer circle of the evolutionary tree showed the relative abundance of each genus in different treatments. The legend on the right shows the phyla corresponding to the genera, and on the left is the treatment information. (g, h) The treatment-induced microbial distribution and abundance at the phylum level. (i) Genus-level heatmaps.

Note: M1: mesophilic stage 1. T: thermophilic stage. M2: stable stage (mesophilic stage 2). B0: control group (feedstock material + rice husks); B1: B0 + biochar pyrolysed at 450 °C; B2: B0 + biochar pyrolysed at 550 °C. B3: B0 + biochar pyrolysed at 650 °C. The size of each circle in the figure changed depending on the absolute value of the correlation, and the colour changed with positive or negative correlation. (a) The relative abundance and distribution of the 10 most annotated gene functions in each sample in the three databases (*cbbL, cbbM* and *nifH*). (b) Correlations among the physicochemical properties of the compost heap and the microbial diversity, with a colour gradient denoting Spearman's correlation coefficients. B-SSA: specific surface area of biochar. B–C/N: C/N ratio of biochar. ST: compost temperature. (c) Correlations among the physicochemical properties of the compost heap and the expression level of OTUs (by the Partial Mantel test) in the three databases (*cbbL, cbbM* and *nifH*). (d) Correlations among the physicochemical properties of the compost heap and the results of the microbial functional gene annotation (by the Mantel test). The permutation was set to 999 iterations.

## Declaration of competing interest

The authors declare that they have no known competing financial interests or personal relationships that could have appeared to influence the work reported in this paper.

## Appendix A Supplementary data

Multimedia component 4 Multimedia component 4

figs1 figs1

figs2 figs2

figs3 figs3
