# Supplementary material for: The effect of biochar prepared at different pyrolysis temperatures on microbially driven conversion and retention of nitrogen during composting
Source: Heliyon. 2023 Feb 13;9(3):e13698. doi: 10.1016/j.heliyon.2023.e13698 (PMC9976328; doi:10.1016/j.heliyon.2023.e13698)
Supplement: Multimedia component 1 [file mmc1.docx]

**2. Materials and methods**

*2.1 Materials and experimental set-up*

Raw materials for composting were sheep dung (D), mushroom cultivation residues (FR), rice husks, and rice husk biochar prepared at three pyrolysis temperatures. Sheep dung as well as rice husks were provided by the Ecological Sheep Farm of Donglin Village, Chengxiang Town, Taicang City. Mushroom residues were purchased from Four Seasons Edible Fungus Co., Ltd., Chengxiang Town, Taicang City. Biochar was produced by Tianjin Yadell Biomass Technology Co., Ltd., by pyrolysis of rice husks as primary material at different temperatures (450℃, 550℃ and 650℃) under anaerobic conditions for 10 hours. Biochar was passed through a 40-mesh sieve after natural cooling. The basic properties of composting raw materials were showed in Table S1.

The study was conducted at the Ecological Fertilizer Plant in Donglin Village, Chengxiang Town, Taicang City, from July to September 2019 for a total of 43 days. The experiment had a control group and three treatment groups, each in three replicates. First, the feedstock materials were mixed up uniformly according to the normal operation method of the fertilizer plant (the wet weight ratio of sheep dung to mushroom residue was 9:1). Then, the rice husks were added in the ratio 15:100. After that, the composting mixture was divided into four large heaps (containing about 700 kg each) for the four treatments, and then BC prepared at 450°C (B1), 550°C (B2) and 650°C (B3) pyrolysis temperature ^[50]^ was added to the composting mixture in the ratio 35:100 (dry basis) based on our previous research. Upon thorough mixing, each heap was divided into three smaller ones for three replicates of each treatment. The moisture content of the final mixture was kept within 68%±2% w/w ^[101]^. Finally, the prepared composting materials were loaded into separate composting simulation reactors. In addition, control group without BC (B0) contained only composting mixture (feedstock material + rice husks).

The experiment adopted the method of natural ventilation stacking and high-temperature aerobic fermentation, and was carried out in 27.0-L composting simulation reactors (Fig. S1). Circular vents with a diameter of 2 cm were evenly distributed on the bottom, accounting for 1/3 of the bottom area. There was an embedded baffle type outlet and three evenly distributed temperature-measuring holes on the side wall. Every 7 days (or when the temperature of the material exceeded 75℃), the material was mixed thoroughly using a mixing system in the container. The material in each container was weighed after each mixing. This procedure was repeated until the composting was complete (on the 43rd day). There was no adjustment of moisture content of the material during composting.

In addition, compost samples from 12 composting reactors were collected on the 6th, 21st and 42nd day after the start of composting, and labeled as M1 (mesophilic stage 1), T (thermophilic stage) and M2 (mesophilic stage 2, stable stage), respectively.

**2.2 Methods**

*2.2.1 Basic indicators of compost and biochar microstructure*

Three temperature-measuring points were positioned evenly on each reactor. Three 60-cm long thermometers were used to measure the heap temperature at a depth of 30 to 40 cm twice a day (9:00-10:00 am and 3:00-4:00 pm). The ambient air temperature was recorded at the same time.

The compost extract (compost sample was mixed with deionized water at a ratio of 1:10, stirred at 150 r/min for 30 min, followed by centrifugation at 450 g for 10 min; then, the supernatant was collected) was used to obtain the pH and EC value of the compost using a multi-parameter water quality analyzer (WTW InoLab pH/Cond 720, Xylem Analytics, Germany).

The biochar microstructure (secondary electron imaging (SEI)) was obtained by an ultra-high resolution field emission scanning electron microscope (SU8010 FE-SEM, HITACHI, Japan) ^[102]^.

*2.2.2 Nitrogen in compost*

Sampling was done every three days, with 12 mixed samples of 2 kg each collected every time from each reactor. Each mixed sample was composed of five random subsamples in each reactor. Each sample was divided into four parts. The first part was dried at 105℃ to determine moisture content. The second part was used to make the compost extract, and the supernatant was collected and stored at -18℃ for analyses. The contents of total carbon (TC), total nitrogen (TN), ammonium nitrogen (NH_4_^+^-N) and nitrate nitrogen (NO_3_^-^-N) of the compost extract were obtained by using a continuous flow analyzer (Skalar San++, Skalar Analytical, Netherlands) ^[7, 103]^. The third part was used to prepare the air-dried compost sample, crush it and pass it through a 100-mesh sieve, and then burn in a muffle furnace at 550℃ to determine the ash content on the 1^st^ and the last (43^rd^) day of composting. The fourth part of each sample was extracted by chloroform fumigation method to determine the soil microbial biomass nitrogen (MBN) in the 0.5 mol/L K_2_SO_4_ extract stored at -18°C until analysis ^[104]^.

The nitrogen loss rate (NLR) was calculated on the premise that the absolute amount of ash content during the composting process was unchanged:

NLR (%) = (N_1_-H_1_/H_43_ ×N_43_)/N_1_ × 100%, whereby N_1_ and N_43_ were the mass fractions of total nitrogen on the 1^st^ and 43^rd^ day of composting, respectively, and H_1_ and H_43_ were the mass fractions of the ash on the 1^st^ and 43^rd^ day of composting, respectively. The mass fractions were based on dry matter (%) ^[46,50]^.

Microbial biomass nitrogen (MBN) = (TN in fumigated fresh compost sample - TN in non-fumigated sample) *2.22 ^[95]^.

Organic nitrogen (ON) = Total nitrogen (TN) - nitrate nitrogen (NO_3_^-^-N) - ammonium nitrogen (NH_4_^+^-N).

*2.2.3 Release of nitrogen-containing gases during composting*

The ammonia collection device (ACD) was composed of an annular groove-shaped base and a transparent polymethyl methacrylate cylinder. The cylinder was 8 cm high, the inner and outer diameters of the base were 18 cm and 22 cm, and the groove depth was 3 cm. The ACD cylinder was 20 cm in diameter and 30 cm in height. Although absorption of ammonia by boric acid is very quick, there was a vent on the top of the ACD cylinder, which was connected to a latex hose (inner diameter 5 mm, and with a valve that would open when the pressure in the device reached 1.5 times the atmospheric pressure) to balance the air pressure. The base was placed on the surface of the compost mixture in the reactor, and the base and the surface of the compost mixture were kept leveled with the ground. A 1-2 cm water layer was injected into the groove (to ensure a better sealing effect), and then the ACD cylinder was fitted into the groove. The water layer was removed at each sampling and then re-established afterwards (Fig. S1).

When the composting started, we placed a 250-mL glass beaker containing 50 mL of 2% (w/v) boric acid solution as absorbent into the above ACD device, and observed the color change of the boric acid solution in real time. When the color of the solution changed from red to green, it was replaced with fresh boric acid absorption solution. The replaced boric acid solution was immediately back-titrated with 0.05 mol/L dilute sulfuric acid (analytical grade, nitrogen-free, ρ=1.84 g·mL^-1^). The time of each replacement of the boric acid solution and the amount of dilute sulfuric acid used in back-titration were recorded.

The 2% w/v boric acid absorption solution was prepared as follows: 20.0 g boric acid was dissolved in 950 mL distilled water, and 20 mL of the mixed indicator (0.099 g bromocresol green and 0.066 g methyl red dissolved in 100 mL of 95% v/v ethanol) was added, and mixed well. An aliquot of 0.1 mol/L sodium hydroxide solution was added dropwise until the solution appeared reddish purple (pH about 4.5), and was then diluted with distilled water to 1-L.

Ammonia emission rate (ERA, mg·kg^-1^·d^-1^) was the amount of the released NH_3_ (*A_t_*) collected in the ACD cylinder by the boric acid absorption solution per unit of composting mixture mass per day, multiplied by 25.16 (the cross-sectional area ratio of the compost reactor and the ACD cylinder). The cumulative amount of NH_3_ emission (CEA, mg·kg^-1^) was the sum of the daily NH_3_ emission amounts, i.e., *CEA* = *A_1_*+ *A_2_*+…+*A_t_*_,_ where *A_t_* was a single-day NH_3_ emission amount (mg·kg^-1^), and *t* was the number of composting days (d).

The nitrous oxide collection device (NCD) was composed of 1-cm-thick polyvinyl chloride (PVC) plastic sheets welded together to make a square-shaped grooved base and a PVC box. The base was 8 cm high, and the groove depth was 3 cm. The length, width and height of the PVC box were 20 cm, 20 cm and 30 cm, respectively. There was a vent (with a self-sealing stopper) in the middle of the box on one side at about 2/3 height (Fig. S1).

The base was placed on the surface of the composting mixture in the reactor, keeping the base as well as the surface of the composting mixture leveled with the ground. A 1-2 cm water layer was injected into the groove (to ensure a better sealing effect), and then the PVC box was inserted into the groove. At 0, 5, 10, and 15 min after the NCD was set up, a 100-mL N_2_O sample was withdrawn with a syringe, transferred to a 100-mL aluminum foil gas-sampling bag comprising a self-sealing gas valve. The nitrous oxide samples were collected every 2 days until the composting was completed, and the sampling time was 9:00-11:00 am. The N_2_O concentration was determined by a gas chromatograph (Agilent 7890 B, Agilent Technologies Inc., California, USA).

Nitrous oxide emission rate (ERN, mg·kg^-1^·d^-1^) =*ρ*V* (dC/dt)*273*16*24/((273+T)*m),* where *ρ* was the density of N_2_O under the standard conditions (1.978 kg·m^-3^). *V*: the volume of the sampling box, m^3^. *dC/dt*: the concentration change rate of the gas in the sampling box over time. *T* was the temperature of the composting mixture (℃). *16*: the cross-sectional area ratio of the compost reactor and the NCD. *24*: 24 hours a day. *m*: the mass of dry matter of the composting mixture (kg).

The cumulative amount of N_2_O emission (CEN, mg·kg^-1^): the average value of ERN at two adjacent sampling time points was multiplied by the duration of the period between the sampling times to obtain the cumulative amount of N_2_O emissions in a certain period of time. By summation over successive time periods, the cumulative amount of N_2_O emissions was obtained.

*2.2.4 Microbial community structure*

To explore whether the addition of biochar could improve the fixation of carbon and nitrogen in the entire system by changing the diversity of autotrophic microorganisms and microorganisms with nitrogen fixation function. Collected sheep dung (D) and mushroom cultivation residues (FR) were sampled before the start of composting. All the collected samples were transferred to the sequencing company in liquid nitrogen for analysis of related carbon fixation (*RuBisCO*) genes (ribulose-1,5-biphosphate carboxylase/oxygenase genes: *cbbL, cbbM*) and nitrogen fixation (dinitrogenase-reductase genes: *nifH*) genes.

The specific primers with barcode or the fusion primers with dislocation of the bases were synthesized according to the designated sequencing regions. The primers for *cbbL, cbbM* and *nifH* were 595F/1387R, 490F/974R and PolF/PolR, respectively ^[105]^. A TransStart FastPfu DNA (TransGen AP221-02) polymerase 20 μL reaction system contained 5×FastPfu buffer 4 μL, 2.5 mmol·L^-1^ dNTPs 2 μL, forward primer (5 μmol·L^-1^) 0.8 μL，reverse primer (5 μmol·L^-1^) 0.8 μL, FastPfu polymerase 0.4 μL, template DNA 10 ng, and ddH_2_O added to make up the total volume of 20 μL.

The *cbbL* gene PCR was performed as follows: 94°C, 3 min, 1 cycle; 94°C, 30 s, 25 cycles; 58 to 48°C, 30 s, 1 cycle; 72°C, 45 s, 1 cycle; 94°C, 20 s, 48°C, 20 s, 72°C, 45 s, 10 cycles; and 72°C, 7 min, 1 cycle. The amplification of the *cbbM* gene was performed under the same conditions, but the annealing temperature was lowered from 62 to 52°C. PCR (*nifH)* was performed with an initial denaturation of 95°C for 5 min, followed by 94°C 1 min, 72°C 2 min, 30 cycles, and a final 5-min extension at 72°C. The PCR products were recovered using an AxyPrep DNA Gel Recovery Kit (Axygen Scientific Inc., Silicon Valley, USA), eluted with Tris-HCl, and detected by 2% w/w agarose gel electrophoresis. The PCR products were quantified by a QuantiFluor™ blue fluorescence quantification system (Promega Corporation, Madison, USA), followed by high-throughput sequencing. The platform for *cbbL* was Roche 454 (Roche 454 Life Sciences, Branford, CT, USA), whereas for *cbbM* and *nifH* it was Illumina MiSeq 2*300 bp (Allwegene Technology Co., Ltd., Beijing, China; Illumina, San Diego California, USA). All sequences were divided into OTUs, and bioinformatics analysis was performed on OTUs with 97% similarity ^[103,106]^.

Besides, we conducted a statistical analysis of bioinformatics for OTUs at a similar level of 97%.

*2.2.5 Statistics*

Statistical analyzes were performed using Microsoft Excel 2016, SPSS Statistics 20.0 (IBM, Armonk, New York, USA) and R package vegan (v2.5-5, v2.5-6, v1.0.12). Adobe Illustrator CC 2017, GraphPad Prism (v8.0.1) and R packages (pheatmap v1.0.12, ggplot2 v3.2.1, ggord v1.15, and corrplot v0.84) were used to draw figures. In addition, FAPROTAX (v1.2.2) was used for the microbial community function prediction (Guillot and Rousset, 2013; Kraemer et al., 2020; Oksanen et al., 2020; Sedgwick, 2014; Shi et al., 2019; Wickham et al., 2020).

The average value of all parameters was taken from the three replicates, and the standard error was calculated. The data were analyzed by the multiple *t*-test (p≤0.05). Correlation analysis used ANOSIM, Mantel tests and Spearman's rank correlation coefficients. The multi-sample clustering tree was constructed using the unweighted pair group method with arithmetic means (UPGMA), and PCA (principal component analysis) was based on OTUs at 97% similarity ^[93,107]^.

*2.3 Availability of data and materials*

The complete sequencing data sets have been deposited in the NCBI Sequence Read Archive (SRA) database under the accession number PRJNA688812 (*cbbL*), PRJNA68862 (*cbbM*) and PRJNA688639 (*nifH*).
