# Supplementary material for: The effect of biochar prepared at different pyrolysis temperatures on microbially driven conversion and retention of nitrogen during composting
Source: Heliyon. 2023 Feb 13;9(3):e13698. doi: 10.1016/j.heliyon.2023.e13698 (PMC9976328; doi:10.1016/j.heliyon.2023.e13698)
Supplement: Multimedia component 2 [file mmc2.docx]

*3.1.1 Basic indicators and structure of biochar*

It could be seen from the secondary electron images (SEI) magnified 5000 times that there were obvious differences in the structure of rice husk biochar formed at three different pyrolysis temperatures. The surface of biochar became smoother with the increased pyrolysis temperature from 450 to 550℃, and the carbon frame structure of the raw material became clearer. In addition, the pore walls of the biochar structure gradually merged, and formed penetrating pits on the pore walls, which increased the porosity and made the pore structure more uniform. Therefore, in this study, the structure of biochar formed at 450°C (BC450) was significantly different from that produced at 550°C (BC550), but this difference gradually weakened when the pyrolysis temperature rose from 550°C to 650°C.

The specific surface area (SSA) increased with an increase in pyrolysis temperature (Table S1), and the micropores and mesopores were more abundant in rice husk biochar formed at 550 ℃ and 650℃ (BC650) than in BC450. The complexity of the pore structure increased with the pyrolysis temperature (Fig. S2).

The contents of total carbon (B-TC) and total nitrogen (B-TN) in biochar decreased significantly as the pyrolysis temperature rose, whereas the ratio of carbon to nitrogen (B-C/N) in biochar significantly increased (p≤0.05). The order of TN was rice husk > BC450 > BC550 > BC650 (p≤0.05), that of and C/N was the opposite (rice husk < BC450 < BC550 < BC650), whereas the order of TC was BC450 = rice husks > BC550 > BC650 (p≤0.05) (Table S1).

*4.1 The effect of biochar prepared at different pyrolysis temperatures on composting*

Biochar is a type of amorphous carbon with the turbostratic microcrystalline structure. Biochar retains the basic characteristics of the microstructure of its raw materials (Laine et al., 1991; Nguyen et al., 2010; Yoo et al., 2018). From the SEI of biochar, we could clearly see the honeycomb structure (Fig. S2), mimicking the basic structural characteristics of the rice husks. However, due to the removal of volatile organic compounds in the pyrolysis process, a porous honeycomb structure with micropores, mesopores and macropores was formed (Hossain et al., 2011). The surface of biochar particles gradually became smooth, with clear outlines (Fig. S2). The order based on total carbon content was BC450 < BC550 < BC650 (p≤0.05) (Table S1).

Micropores (pore width of 0-2 nm) contributed significantly to the specific surface area and adsorption capacity of biochar, whereas mesopores (pore width of 2-300 nm) had a greater impact on pore volume (Lu et al., 2020; Nguyen et al., 2010; Wang et al., 2018). In this study, the porous structure comprised mainly mesopores and micropores; the complexity of the porous structure and specific surface area of biochar increased as the pyrolysis temperature rose, with relatively few micropores and mesopores in BC450, and more of them in BC550 and BC650 (Fig. S2). The porous structures of the biochar may be caused by the hydrolysis of polysaccharides and the cleavage of lignin during low-temperature pyrolysis, whereas decomposition of organic matter (polysaccharides, cellulose, etc.) intensified with an increase in pyrolysis temperature, and promoted the release of nitrogen- and carbon-containing gases. The original biomass structure disappeared, leaving abundant pores and porous carbon framework structures such as carbonized lignin (Li et al., 2016; Lu et al., 2020).
