# Supplementary material for: The effect of biochar prepared at different pyrolysis temperatures on microbially driven conversion and retention of nitrogen during composting
Source: Heliyon. 2023 Feb 13;9(3):e13698. doi: 10.1016/j.heliyon.2023.e13698 (PMC9976328; doi:10.1016/j.heliyon.2023.e13698)
Supplement: Multimedia component 3 [file mmc3.docx]

The results of ANOSIM analysis showed there was a medium difference (R-value 0.703) in the composition of *cbbL*-containing OTUs among the treatments. No R-value was less than zero. Except for TB1-TB2, M1B2-M1B3, M1B2-TB0 and M2B2-M2B3, the R-values of all the other treatment pairs were greater than 0.25.

The overall R-value of *cbbM*-containing OTUs was 0.751, which showed there was a medium to big difference among the treatments. Except for M2B1-M2B2, TB3-M2B2, M2B1-M2B3, M2B2-M2B3 and TB3-M2B3, the R-values of the remaining treatment pairs were all greater than 0.25.

There was a big difference in the composition of *nifH*-containing OTUs among the treatments (the overall R-value was 0.856). Except for M2B2-M2B3 and M1B0-M1B1, the R-values of the other treatment pairs were all greater than 0.25. This analysis indicated the microbial community composition in different treatments might have been similar during the composting process. In particular, the community composition of B2 and B3 was strikingly similar (Table S3).
