# Supplementary material for: The effect of biochar prepared at different pyrolysis temperatures on microbially driven conversion and retention of nitrogen during composting
Source: Heliyon. 2023 Feb 13;9(3):e13698. doi: 10.1016/j.heliyon.2023.e13698 (PMC9976328; doi:10.1016/j.heliyon.2023.e13698)
Supplement: Multimedia component 4 [file mmc4.docx]

**Table S1 General OTUs and taxonomic information**

| Genes | OTUs | Phyla | Classes | Orders | Families | Genera |
| --- | --- | --- | --- | --- | --- | --- |
| *cbbL* | 800 | 10 | 15 | 28 | 42 | 62 |
| *cbbM* | 6112 | 25 | 43 | 98 | 194 | 385 |
| *nifH* | 1089 | 19 | 34 | 72 | 119 | 190 |

**Table S2** **OTUs cluster analysis（genus level）**

| Genes | All genera detected |
| --- | --- |
| *cbbL* | *Acidiferrobacter, Acidithiobacillus, Acinetobacter, Actinoalloteichus, Alkalilimnicola, Alkalispirillum, Allochromatium, Arthrobacter, Aspergillus, Asterococcus, Bacillus、Caenispirillum、Candida, Clostridium, Cohnella, Cupriavidus, Ectothiorhodospira, Endothiovibrio、Escherichia, Gillisia, Guyparkeria, Halorhodospira, Halothiobacillus, Hydrogenophaga, Hydrogenovibrio, Kluyveromyces, Lactobacillus, Lichtheimia, Marichromatium, Mesorhizobium, Methanobrevibacter, Methylocaldum, Methylococcus, Methylonatrum, Methylophaga, Mycobacterium, Nitrobacter, Nitrosomonas, Nitrosospira, Nostoc, Paenibacillus, Pelotomaculum, Phaeobacter, Pichia, Pseudomonas, Rhizopus, Rhodobacter, Rhodovulum, Romboutsia, Sphingobacterium, Sulfuricaulis, Sulfurifustis, Sulfuritortus, Thioalkalivibrio, Thiobacillus, Thiocapsa, Thiocystis、Thiohalobacter, Thiohalomonas, Thiomicrorhabdus, unidentified, Ureibacillus* |
| *cbbM* | *Acetobacter, Acetobacterium, Acholeplasma, Achromobacter, Acidihalobacter, Acidipropionibacterium, Acidithiobacillus, Acidovorax, Acinetobacter, Actinoalloteichus, Actinomadura, Actinoplanes, Actinopolyspora, Acutalibacter, Advenella, Aerococcus, Aeromonas, Agrobacterium, Agrococcus, Agromyces, Alcaligenes, Alcanivorax, Algoriphagus, Alistipes, Alkalilimnicola, Alkalitalea, Allochromatium, Allokutzneria, Alteromonas, Aminobacter, Amycolatopsis, Anthracocystis, Aquabacterium, Aquitalea, Archangium, Arsenicicoccus, Arthrobacter, Asticcacaulis, Azoarcus, Azorhizobium, Azospirillum, Azotobacter, Bacillus, Bacteroides, Barnesiella, Bdellovibrio, Bifidobacterium, Blautia, Bordetella, Bosea, Brachybacterium, Bradyrhizobium, Brassica, Breoghania, Brevibacillus, Brevibacterium, Brevundimonas, Burkholderia, Butyrivibrio, Campylobacter, Candidatus_Acetothermum, Candidatus_Hamiltonella, Candidatus_Koribacter, Candidatus_Paracaedibacter, Candidatus_Thioglobus, Carboxydocella, Cardiobacterium, Castellaniella, Catenulispora, Caulobacter, Cellulomonas, Cellvibrio, Chelatococcus, Christensenella, Chromobacterium, Chryseobacterium, Chryseolinea, Citrobacter, Clavibacter, Cloacibacillus, Clostridium, Colletotrichum, Collimonas, Collinsella, Comamonas, Corallococcus, Corynebacterium, Crenobacter, Croceicoccus, Cryobacterium, Cupriavidus, Cutibacterium, Dactylosporangium, Defluviimonas, Defluviitoga, Dehalobacter, Deinococcus, Delftia, Desulfallas, Desulfarculus, Desulfitobacterium, Desulfobulbus, Desulfomicrobium, Desulfosporosinus, Desulfotomaculum, Desulfovibrio, Desulfurispirillum, Desulfuromonas, Dickeya, Dietzia, Dokdonella, Dyella, Ectothiorhodospira, Edwardsiella, Eggerthella, Elusimicrobium, Endothiovibrio, Enterobacter, Enterococcus, Erythrobacter, Escherichia, Ethanoligenens, Eubacterium, Exiguobacterium, Faecalibacterium, Faecalitalea, Fermentimonas, Ferrimonas, Fibrella, Filifactor, Flaviflexus, Flavisolibacter, Flavobacterium, Flavonifractor, Fonticula, Friedmanniella, Gaeumannomyces, Gardnerella, Gemmatirosa, Gemmobacter, Geobacillus, Geobacter, Georgenia, Glaesserella, Glutamicibacter, Gordonia, Gordonibacter, Haliangium, Halioglobus, Halomonas, Haloterrigena, Halothiobacillus, Heliobacterium, Herbaspirillum, Herbinix, Hungateiclostridium, Hydrogenophaga, Hydrogenovibrio, Hymenobacter, Immundisolibacter, Indioceanicola, Intestinimonas, Janibacter, Janthinobacterium, Jatrophihabitans, Kerstersia, Ketobacter, Klebsiella, Kluyveromyces, Kocuria, Kozakia, Kribbella, Kytococcus, Labrenzia, Lachnoclostridium, Lacinutrix, Lactobacillus, Lautropia, Leifsonia, Leptothrix, Limnochorda, Limnohabitans, Luteibacter, Luteimonas, Luteitalea, Lysinibacillus, Lysinimonas, Lysobacter, Macrococcus, Magnetospira, Magnetospirillum, Magnetovibrio, Maribacter, Marinactinospora, Marinobacter, Marinobacterium, Marinospirillum, Marinovum, Marmoricola, Martelella, Massilia, Mastacembelus, Megasphaera, Meiothermus, Melissococcus, Melittangium, Mesorhizobium, Methanobrevibacter, Methanoculleus, Methanothrix, Methylobacterium, Methylocaldum, Methylocella, Methylococcus, Microbacterium, Microbulbifer, Micrococcus, Micromonospora, Minicystis, Modestobacter, Moritella, Mucilaginibacter, Mycetocola, Mycobacterium, Mycolicibacterium, Myxococcus, Neoasaia, Neorhizobium, Nitratireductor, Nitrosococcus, Nitrosomonas, Nitrosospira, Nitrospira, Nitrospirillum, Nocardia, Nocardioides, Nonomuraea, Nostoc, Novosphingobium, Oblitimonas, Oceanimonas, Ochrobactrum, Odoribacter, Oligella, Olsenella, Oscillibacter, Ottowia, P2virus, Paenalcaligenes, Paenibacillus, Pandoraea, Pannonibacter, Pantholops, Parabacteroides, Paraburkholderia, Paracoccus, Paraprevotella, Parolsenella, Parvibaculum, Paucibacter, Paucimonas, Pelagibacterium, Pelobacter, Pelosinus, Pelotomaculum, Petrimonas, Phaeobacter, Phaeospirillum, Phenylobacterium, Phoenicibacter, Phreatobacter, Phycicoccus, Phycisphaera, Pisidia, Planctomyces, Planococcus, Plantactinospora, Pleomorphomonas, Pluralibacter, Podospora, Polymorphum, Polynucleobacter, Pontibacter, Porphyrobacter, Prevotella, Proteiniphilum, Pseudoalteromonas, Pseudodesulfovibrio, Pseudomonas, Pseudoxanthomonas, Psychrobacter, Psychromicrobium, Pusillimonas, Ralstonia, Ramlibacter, Rhizobacter, Rhizobium, Rhodanobacter, Rhodobaca, Rhodobacter, Rhodococcus, Rhodomicrobium, Rhodopseudomonas, Rhodothermus, Rhodovulum, Roseburia, Roseibacterium, Roseovarius, Rothia, Rubrivivax, Ruminococcus, Ruthenibacterium, Saccharopolyspora, Salinisphaera, Salipiger, Salmonella, Sanguibacter, Selenomonas, Serinicoccus, Serratia, Shewanella, Sinorhizobium, Sorangium, Sphaerobacter, Sphaerochaeta, Sphingobacterium, Sphingobium, Sphingomonas, Sphingopyxis, Spirosoma, Sporolactobacillus, Stackebrandtia, Stenotrophomonas, Streptococcus, Streptomyces, Sulfitobacter, Sulfuricaulis, Sulfurifustis, Sulfuritalea, Sulfuritortus, Sulfurivermis, Symbiobacterium, Synechococcus, Tannerella, Terrabacter, Tessaracoccus, Thalassococcus, Thauera, Thermaerobacter, Thermanaerovibrio, Thermoanaerobacter, Thermoanaerobacterium, Thermobacillus, Thermobifida, Thermobispora, Thermoclostridium, Thermomonospora, Thermoproteus, Thermosynechococcus, Thermotoga, Thermus, Thioalkalivibrio, Thiobacillus, Thiodictyon, Thioflavicoccus, Thiohalobacter, Thiohalomonas, Thiomicrospira, Thiomonas, Trueperella, Tsukamurella, Turicibacter, unidentified, Variovorax, Verminephrobacter, Verticillium, Vibrio, Virgibacillus, Vogesella, Vulgatibacter, Xanthomonas, Yangia, Yersinia, Zhongshania, Zobellella* |
| *nifH* | *Acetobacter, Achromobacter, Acidiphilium, Actinoplanes, Acutalibacter, Aeromonas, Alcanivorax, Alicycliphilus, Altererythrobacter, Ammonifex, Amycolatopsis, Anaeromyxobacter, Archangium, Aspergillus, Aureimonas, Azoarcus, Azohydromonas, Azorhizobium, Azospira, Azospirillum, Azotobacter, Bacillus, Bacteroides, Bdellovibrio, Blautia, Bordetella, Bradyrhizobium, Castellaniella, Caulobacter, Cellulosilyticum, Chelatococcus, Chlorella, Christensenella, Chromobacterium, Clostridium, Cohnella, Comamonas, Coraliomargarita, Corynebacterium, Cupriavidus, Cutibacterium, Dechloromonas, Deinococcus, Desulfitobacterium, Desulfobacter, Desulfobotulus, Desulfobulbus, Desulfomicrobium, Desulfonatronum, Desulfovibrio, Desulfurispirillum, Desulfuromonas, Devosia, Egibacter, Erysipelothrix, Escherichia, Ethanoligenens, Euzebya, Faecalibacterium, Fibrobacter, Filimonas, Flavonifractor, Frankia, Fretibacterium, Geobacter, Geosporobacter, Geovibrio, Gluconobacter, Glycocaulis, Gordonia, Gordonibacter, Haemophilus, Halioglobus, Halomonas, Halorhodospira, Herbaspirillum, Hungateiclostridium, Immundisolibacter, Klebsiella, Komagataeibacter, Kurthia, Lachnoclostridium, Lactobacillus, Limnochorda, Lodderomyces, Luteimonas, Luteitalea, Lysobacter, Magnetospirillum, Marinobacter, Massilia, Meiothermus, Melittangium, Mesorhizobium, Methanosarcina, Methanothermobacter, Methylobacter, Methylobacterium, Methylocaldum, Methylomusa, Microbulbifer, Micromonas, Micromonospora, Mitsuaria, Modestobacter, Monascus, Mycobacterium, Nitratireductor, Nitrosococcus, Nitrospirillum, Nocardia, Nonomuraea, Olsenella, Orrella, Paenibacillus, Pandoraea, Paraburkholderia, Paraprevotella, Pediococcus, Pelobacter, Pelosinus, Petrimonas, Phascolarctobacterium, Phreatobacter, Pichia, Plantactinospora, Porphyromonas, Pseudacidovorax, Pseudoclostridium, Pseudodesulfovibrio, Pseudogulbenkiania, Pseudomonas, Pseudoxanthomonas, Ralstonia, Ramlibacter, Raoultella, Rhizobium, Rhizopus, Rhodoferax, Rhodospirillum, Rhodothermus, Rhodovulum, Roseburia, Rubrivivax, Ruficoccus, Ruminococcus, Saccharomycopsis, Salinibacter, Serratia, Shinella, Sinorhizobium, Sorangium, Sphaerochaeta, Spiribacter, Starkeya, Stenotrophomonas, Streptacidiphilus, Streptomyces, Sulfuricaulis, Sulfuricurvum, Sulfuritortus, Sulfurivermis, Symbiobacterium, Tannerella, Terriglobus, Tessaracoccus, Thauera, Thermanaerovibrio, Thermoascus, Thermobifida, Thermoclostridium, Thermomonospora, Thermomyces, Thioalkalivibrio, Thiodictyon, Thiohalobacter, Thiolapillus, Thiomonas, Treponema, Trichodesmium, unidentified, Vibrio, Vogesella, Xanthobacter, Xanthomonas, Xenorhabdus, Yersinia, Zea, Zoogloea* |

**Table S3 ANOSIM analysis**

| R-value | *cbbL* | *cbbM* | *nifH* |
| --- | --- | --- | --- |
| (0.75 to 1) | M1B0-TB3, M1B0-M2B1, M1B0-M2B2, M1B0-M2B3, M1B1-TB0, M1B1-TB1, M1B1-TB3, M1B1-M2B3, M1B1-D, M1B3-TB1, M1B3-TB3, M1B3-M2B2, M1B3-M2B3, TB0-TB1, TB0-M2B2, TB0-M2B3, TB0-D, TB1-TB3, TB1-M2B3, TB1-D, TB2-D, TB3-M2B3, TB3-D, M2B1-D, M2B2-D, M2B3-D, M1B1-M1B3, M1B3-D, TB0-TB3, TB2-M2B3, M1B0-TB1, M1B0-TB2, M1B2-D, TB0-M2B1, TB1-M2B2, TB2-M2B2, M1B1-TB2, M1B0-M1B3, M1B2-M2B3, TB2-TB3, M1B1-M2B2, TB3-M2B2, M1B0-M1B2, M1B0-TB0, M1B0-D | M1B0-M1B1, M1B0-M1B3, M1B0-TB1, M1B0-TB2, M1B0-TB3, M1B0-M2B1, M1B0-M2B2, M1B0-M2B3, M1B0-D, M1B1-M1B3, M1B1-TB0, M1B1-D, M1B3-TB0, M1B3-TB1, M1B3-TB3, M1B3-M2B1, M1B3-M2B2, M1B3-M2B3, M1B3-D, TB0-TB1, TB0-TB2, TB0-TB3, TB0-M2B1, TB0-M2B2, TB0-M2B3, TB0-D, TB1-TB3, TB1-D, TB2-D, TB3-D, M2B1-D, M2B2-D, M2B3-D, M1B1-TB3, M1B1-M2B3, M1B1-TB2, M1B1-M2B2, TB1-M2B3, M1B0-TB0, M1B3-TB2 | M1B0-TB0, M1B0-TB1, M1B0-TB3, M1B0-M2B1, M1B0-M2B2, M1B0-M2B3, M1B0-D, M1B0-FR, M1B1-TB1, M1B1-TB3, M1B1-M2B1, M1B1-M2B2, M1B1-M2B3, M1B1-D, M1B1-FR, M1B2-TB1, M1B2-TB3, M1B2-M2B1, M1B2-M2B2, M1B2-M2B3, M1B2-D, M1B2-FR, M1B3-TB1, M1B3-TB3, M1B3-M2B1, M1B3-M2B2, M1B3-M2B3, M1B3-D, M1B3-FR, TB0-TB1, TB0-TB3, TB0-M2B1, TB0-M2B2, TB0-M2B3, TB0-D, TB0-FR, TB1-TB3, TB1-M2B1, TB1-M2B2, TB1-M2B3, TB1-D, TB1-FR, TB2-FR, TB3-M2B1, TB3-M2B2, TB3-M2B3, TB3-D, TB3-FR, M2B1-D, M2B1-FR, M2B2-D, M2B2-FR, M2B3-D, M2B3-FR, D-FR, M1B0-TB2, TB2-M2B1, M1B1-TB0, TB2-D, M2B1-M2B3, M1B0-M1B2, M1B1-M1B2, M1B1-TB2 |
| (0.5 to 0.75) | M1B0-M1B2、M1B0-TB0、  M1B0-D、M1B2-M2B2、  M1B3-TB2、M1B3-M2B1、  TB0-TB2、M1B2-TB3、  TB3-M2B1 | M1B1-M2B1、TB1-M2B2、  TB1-TB2、TB2-TB3、  TB2-M2B1 | M1B0-M1B3、TB2-M2B2、  TB2-M2B3、M1B1-M1B3 |
| (0.25 to 0.5) | M2B1-M2B2、M1B0-M1B1、  M1B2-TB1、M1B2-M2B1、  M1B1-M2B1、M1B1-M1B2、  TB1-M2B1、M2B1-M2B3、  M1B2-TB2、M1B3-TB0、  TB2-M2B1 | TB1-M2B1、TB2-M2B3、  TB3-M2B1、M1B1-TB1、  TB2-M2B2 | M1B2-M1B3、TB2-TB3、  M2B1-M2B2、M1B2-TB0、  TB1-TB2 |
| (0 to 0.25) | TB1-TB2、M1B2-M1B3、  M1B2-TB0、M2B2-M2B3 | M2B1-M2B2、TB3-M2B2 | M2B2-M2B3、M1B0-M1B1 |
| (-1 to 0) | None | M2B1-M2B3、M2B2-M2B3、  TB3-M2B3 | None |

Note: M1: mesophilic stage 1. T-1: the first half of the thermophilic stage. T-2: the second half of the thermophilic stage. M2-1: the first half of the stable stage. M2-2: the second half of the stable stage. B0: control group (feedstock material + rice husks); B1: feedstock material + biochar pyrolysed at 450°C; B2: feedstock material + biochar pyrolysed at 550°C. B3, feedstock material + biochar pyrolysed at 650°C.
