# Supplementary figures and images for: The effect of biochar prepared at different pyrolysis temperatures on microbially driven conversion and retention of nitrogen during composting
Source: Heliyon. 2023 Feb 13;9(3):e13698. doi: 10.1016/j.heliyon.2023.e13698 (PMC9976328; doi:10.1016/j.heliyon.2023.e13698)

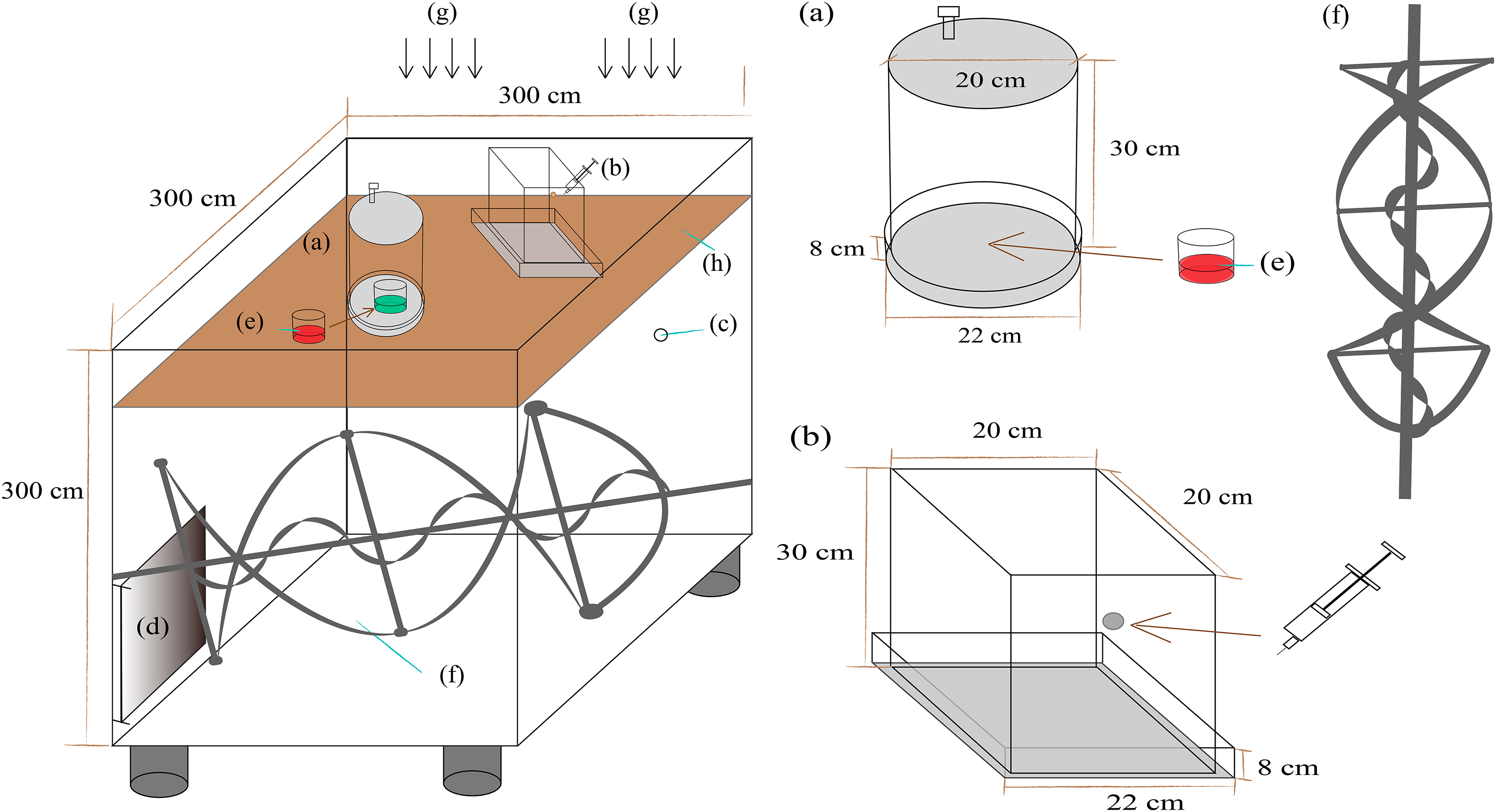

Supplement: figs1 [file mmcfigs1.jpg]

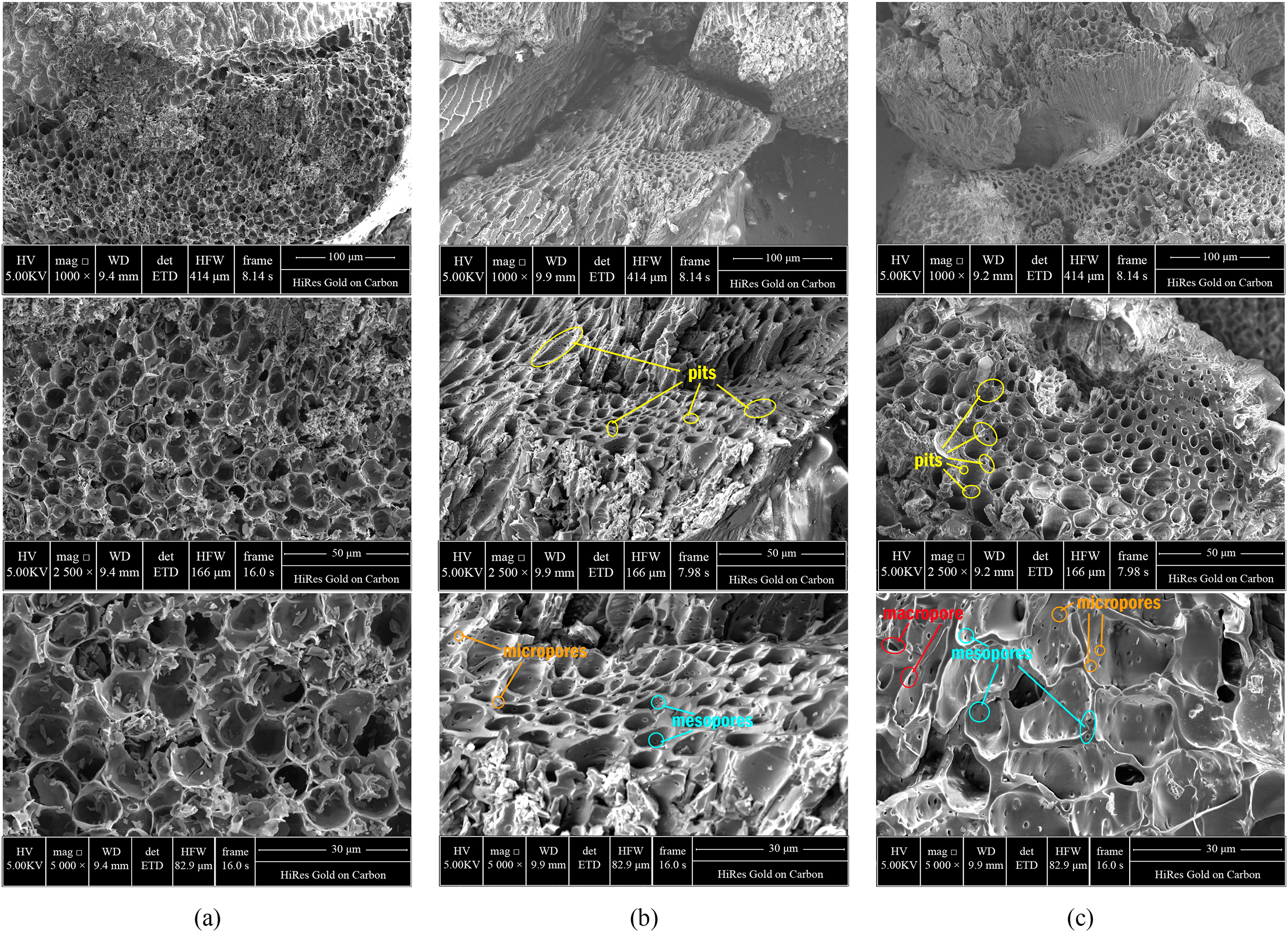

Supplement: figs2 [file mmcfigs2.jpg]

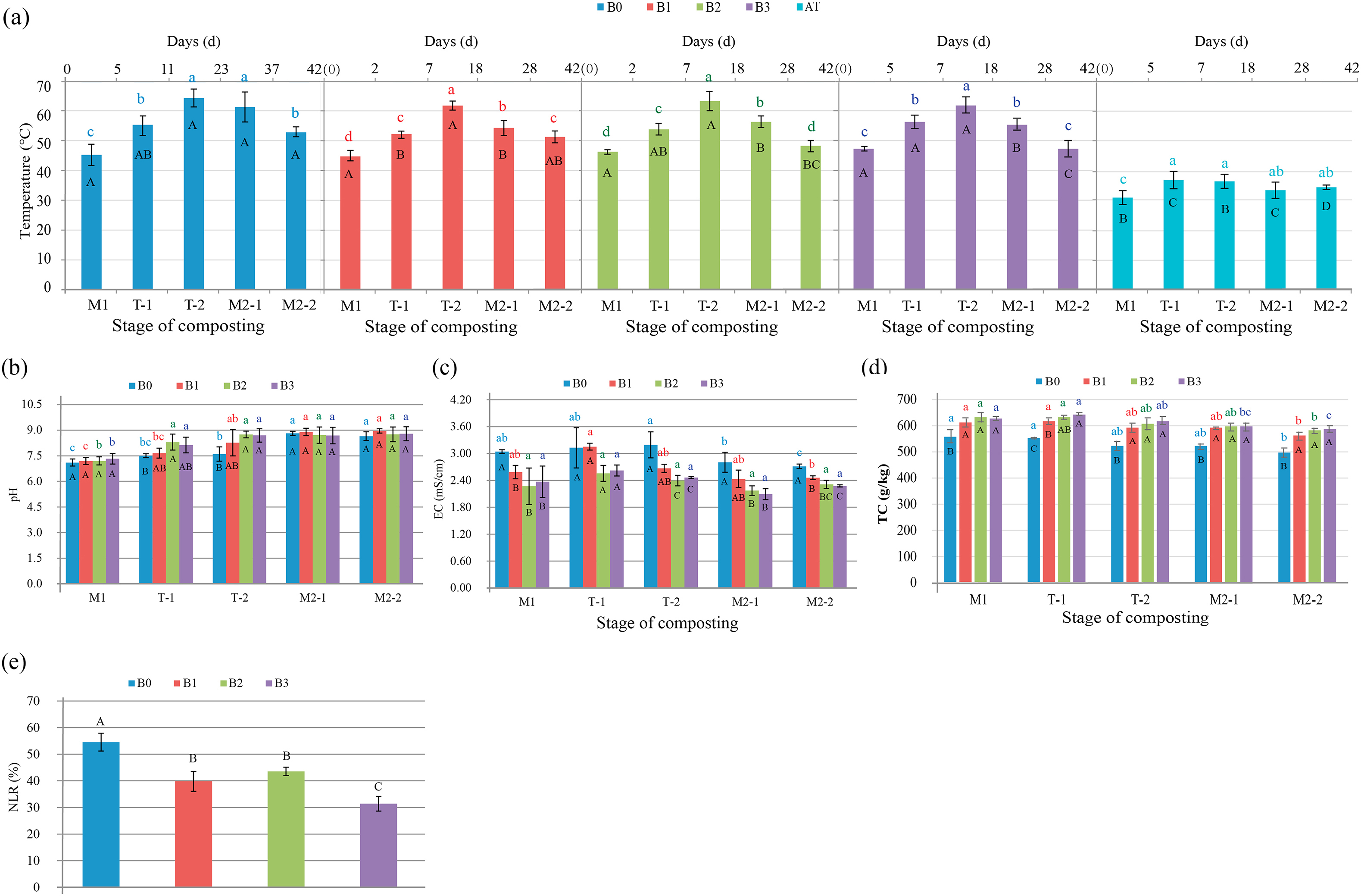

Supplement: figs3 [file mmcfigs3.jpg]
